# Case report: Successful and effective percutaneous closure of a deep femoral artery pseudoaneurysm using proglide device

**DOI:** 10.3389/fsurg.2023.1109243

**Published:** 2023-03-31

**Authors:** Liu Jiaxin, Li Yan, Zhang Sheng, Dong Zhiyi, Wang Jichang, Lu Shaoying

**Affiliations:** ^1^ Department of Vascular Surgery, First Affiliated Hospital of Xi’an Jiaotong University, Xi’an, China; ^2^ Department of General Surgery, Children’s Hospital of Xi’an Jiaotong University, Xi’an, China; ^3^ Department of General Surgery, Yulin Traditional Chinese Medicine Hospital, Yulin, China

**Keywords:** deep femoral artery, pseudoaneurysm, proGlide, angiography, guide wire

## Abstract

A 61-year-old man developed severe swelling in the left lower extremity after interventional embolization of liver tumor. Ultrasound examination showed a pseudoaneurysm and thrombosis in the upper thigh on the left. To recognize the causes and determine the effective therapy, lower extremity arteriography was performed. The results revealed a pseudoaneurysm arised from deep femoral artery. Considering of the size of cavity and symptoms of patient, a new method was tried instead of traditional treatment using PROGLIDE device. Postoperative angiography showed a powerful blocking effect. This case study provide us a specific treatment for pseudoaneurysm, and this method provide us a new therapeutic strategy in clinical practice.

## 1. Introduction

Pseudoaneurysm, an encapsulated hematoma that is connected to the arterial puncture site, is the most common complication during the interventional treatment of cardiovascular disease (1). In clinical, pseudoaneurysm frequently occurs due to a low puncture site and insufficient angiopressure support after catheter removal (2). However, until now, the current therapy for pseudoaneurysms mainly focuses on traditional treatments (3). Here we describe 1 cases of deep femoral artery pseudoaneurysm after interventional embolization of liver tumor.

## 2. Case presentation

**Figure 1:**
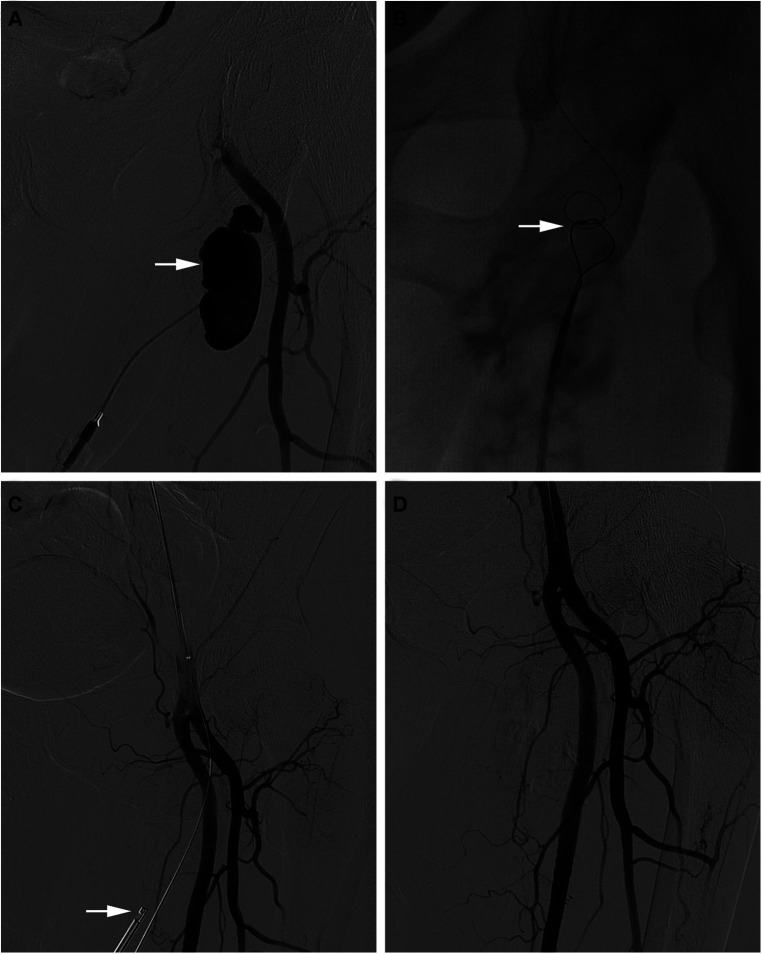
(**A**) Angiography result revealed a 2 cm × 5 cm-sized pseudoaneurysm arising from a branch of the left deep femoral artery. (**B**) The catcher is used to catch the guide wire from the contralateral sheath. (**C**) A PROGLIDE device was used to suture the rupture of the pseudoaneurysm. (**D**) Angiography showed the rupture is completely closed.

A 61-year-old male suffered pain and swelling in her left lower extremity after a interventional operation. He had a medical history of interventional embolization of liver tumors 1 month ago. Vascular ultrasound clearly revealed a pseudoaneurysm connecting with left deep femoral artery with a symbol of mural thrombus within the lumen. Surgery was arranged on the 5nd day. Routine preoperative examination was evaluated prior to the surgery. Angiography result revealed a 2 cm × 5 cm-sized pseudoaneurysm arising from a branch of the left deep femoral artery (Figure 1A). After the placement of 6-F sheath (Terumo, Tokyo, Japan) over pseudoaneurysm sac, then another guide wire was transported into the pseudoaneurysm sac *via* another 6-F sheath over right femoral artery access. The catcher is used to catch the guide wire from the contralateral sheath (Figure 1B). A PROGLIDE device (Abbott Vascular, Redwood City, CA, United States) was used to suture the rupture of the pseudoaneurysm (Figure 1C), and the second angiography showed the rupture is completely closed (Figure 1D). The vital signs and symptoms of patient were stable after the procedure. Without any other complications, she was discharged 2 days after the operation. Informed consent was obtained from the patient for the publication of this study.

## 3. Discussion

Iatrogenic pseudoaneurysm is a vascular complication after interventional examination. In clinical practice, color doppler ultrasound was used to observe characteristic reciprocating signal of blood flow in the aneurysm cavity (4). Considering the serious consequences of the disease such as vascular rupture (5), thromboembolism (6), compression of peripheral nerve tissue (7) and skin tissue necrosis (8). so it is a crucial issue how to deal with pseudoaneurysm. In this case, we suspected that low-position puncture and insufficient compression caused a pseudoaneurysm of deep femoral artery.

We reviewed the relevant literature to find the solution of pseudoaneurysm, the method varies based on size of cavity and symptoms of patients. A cavity <2 cm in diameter that is not extending can be managed conservatively. During conservative procedure, external compression of the puncture can be the first option (9). If fails, multiple embolic materials, including polyvinyl alcohol (PVA) particles, sodium alginate microspheres amd coils, are injected into the aneurysm neck (10). Besides, surgery can be performed when some complication such as swelling, skin necrosis or compression of peripheral nerve tissue occurs (11). The puncture was sutured and the hematoma was removed in the pseudoaneurysm. In this cases, non-surgical treatments such as compression or a simple embolization could not be conducted due to severe pain and oversized pseudoaneurysm cavity. A closure device was used to suture puncture. Compared to other treatments, PROGLIDE device (12) shows an excellent blocking effect, effectively avoiding the trauma of surgical treatment, and also shortening the patient's hospital time.

## Conclusion

Our study demonstrate another important function of PROGLIDE closure device, an excellent blocking effect depend on the combination of the closure device and guide wire was observed. This report describes a specific treatment of pseudoaneurysm, and this method would provide us a new therapeutic strategy in clinical practice.

## Data Availability

The raw data supporting the conclusions of this article will be made available by the authors, without undue reservation.
